# The Ion Channel-Related Gene Signatures Correlated With Diagnosis, Prognosis, and Individualized Treatment in Patients With Clear Cell Renal Cell Carcinoma

**DOI:** 10.3389/fphar.2022.889142

**Published:** 2022-06-01

**Authors:** Zhenpeng Zhu, Zhenchuan Lei, Jinqin Qian, Cuijian Zhang, Yanqing Gong, Guicao Yin, Yifan Li, Xuesong Li, Jian Lin, Liqun Zhou

**Affiliations:** ^1^ National Urological Cancer Center, Department of Urology, Institute of Urology, Clinical Research Cooperation Network of Urology of the Peking University First Hospital, The Peking University First Hospital, Peking University, Beijing and the Affiliated Hospital of Yangzhou University, Yangzhou University, Yangzhou, China; ^2^ School of Biomedical Sciences, Heart and Vascular Institute and Li Ka Shing Institute of Health Sciences, The Chinese University of Hong Kong, Hong Kong, China

**Keywords:** clear cell renal cell carcinoma, ion channel-related genes, prognosis, diagnosis, nomogram

## Abstract

**Background:** Early detection and precise prognostic evaluation of clear cell renal cell carcinoma (ccRCC) are crucial for patient life expectancy. Ion channel-related genes (ICRGs) are of great diagnostic and prognostic value as components that maintain the normal structure of the kidney. Therefore, we systematically explored the diagnostic, prognostic, and therapeutic value of ICRGs in ccRCC using the multi-database.

**Methods:** RNA transcriptome profiles and clinical data of ccRCC patients were extracted and integrated from public databases including The Cancer Genome Atlas, ICGC, GEO, and E-MTAB databases. Ion channel-related genes were obtained from the literature collection. The diagnostic signature was performed using the LASSO and SVM-REF analyses. Meanwhile, the prognostic signature was conducted using the LASSO analyses. Molecular subtyping was performed using the ConsensusClusterPlus and the corresponding therapeutic targets were evaluated using the pRRophetic package. In addition, a prognostic nomogram was constructed based on the results of cox regression analyses.

**Results:** We successfully constructed diagnostic signatures for five ICRGs and prognostic signatures for 10 ICRGs with AUC values greater than 0.7, showing good predictive performance. Based on the median risk score, we found that high-risk patients had a significantly worse prognosis. We also divided ccRCC patients into two clusters according to prognostic ICRGs, and there was a significant survival outcome between the two clusters and different sensitivity to diverse clinical therapeutic strategies. Meanwhile, we constructed a nomogram based on clinical molecules and signatures, and its predictive efficacy was better than the signature or the present tumor-node-metastasis staging system.

**Conclusion:** In this study, we established useful signatures for early detection, prognosis evaluation, and individualized treatment for ccRCC. Moreover, KCNJ16 deserves to be explored comprehensively in the future.

## Introduction

As the major histological subtype of renal cell carcinoma (RCC), clear cell renal cell carcinoma (ccRCC) is one of the common genitourinary tumors with high morbidity and mortality rate, accounting for 2% of the global cancer burden (Turajlic et al., 2018; Siegel et al., 2021). Patients with ccRCC diagnosed at an early stage are often intended for a preferred outcome, while once metastasis occurs, their survival expectation decreased significantly (Cohen and McGovern 2005). In clinical practice, medical imaging is the primary tool for diagnosing, yet it has little effect on early-stage ccRCC. Moreover, it seems insufficient to predict outcomes of ccRCC by tumor-node-metastasis (TNM) staging alone (Barata and Rini 2017). Hence, early detection of ccRCC and proper assessment of its prognosis remains a challenge for urologists.

As an important site for ion exchange in the body, the kidney is dotted with numerous ion channels. Ion channels play vital roles in substance metabolism, signal transmission, and energy exchanging. In many kidney diseases, alterations in ion channels influence the disease processes (Devuyst 2018; Sudarikova et al., 2021). During the development of kidney cancer, the destruction of a large number of kidney units leads to alterations in ion channels. Meanwhile, multiple ion channels are disrupted and altered during the development of ccRCC (Cecconi and Jäättelä 2014; Moosavi and Elham 2020; Li et al., 2020). Therefore, exploring the alteration of ion channels in the development of ccRCC may facilitate the diagnosis and evaluation and the corresponding treatment.

This study used multi-omics databases to screen and validate ion channel-related genes’ (ICRGs) diagnostic and prognostic roles. We successfully developed corresponding diagnostic and prognostic signatures, and both signatures showed good predictive performances. Furthermore, we molecularly subtyped the samples in TCGA and explored the effects of immunotherapy and drugs on ccRCC patients with two clusters. In conclusion, our study was the first to explore the impact of ICRGs on the diagnosis, prediction, and individualized treatment of ccRCC.

## Materials and Methods

### Data Collection and Processing

**FIGURE 1:**
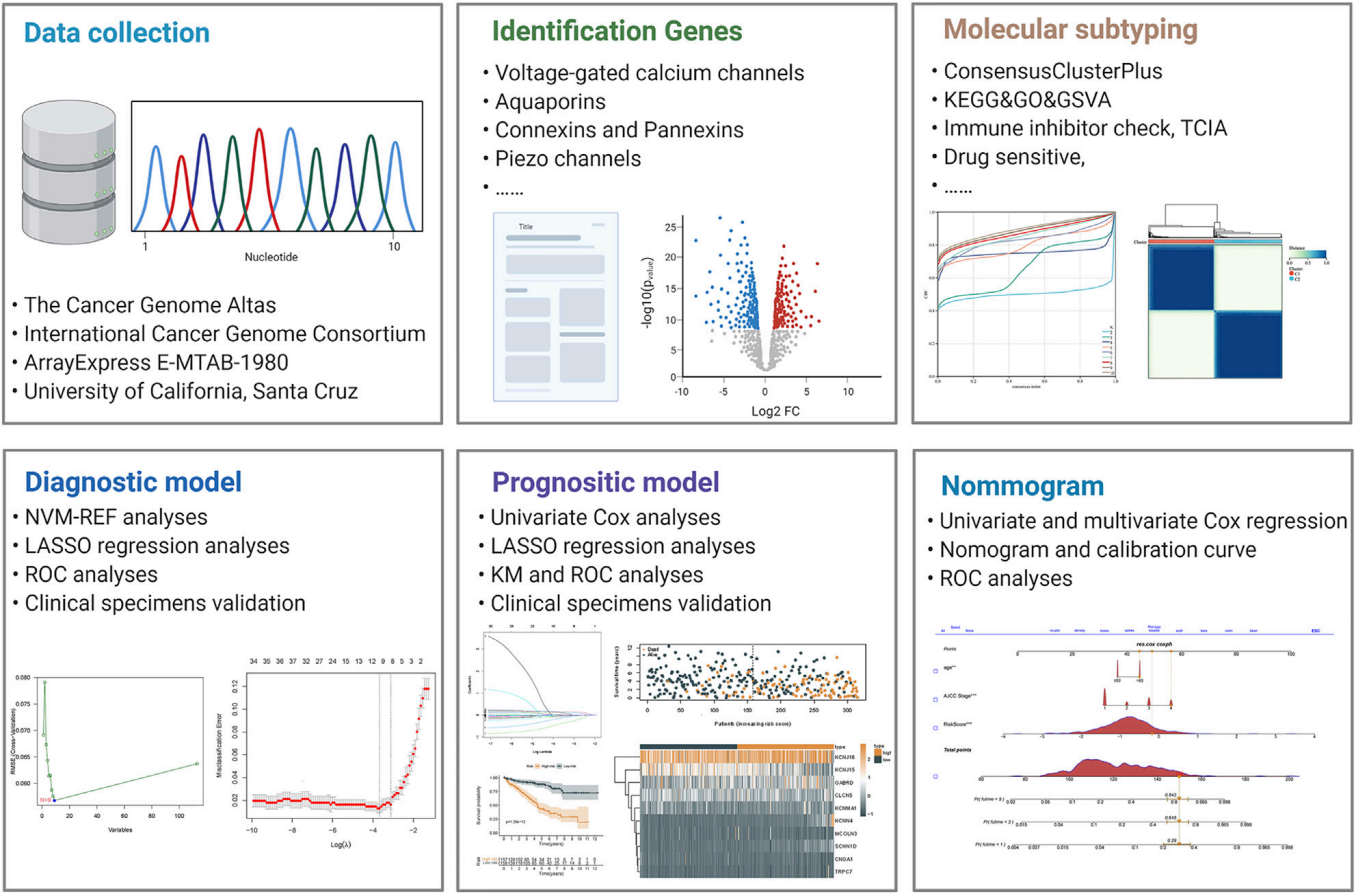
Graphical outline diagram of the entire process of this study.

The mRNA transcriptomic profile and corresponding clinical data of ccRCC samples were downloaded from The Cancer Genome Atlas (TCGA), including 72 normal controls and 539 cancer samples. The detailed information of patients’ information has been showed in the Table 1. Duplicate samples and those with an overall survival time of fewer than 30 days were excluded. Later, when building the LASSO regression model, we retained TCGA samples containing both RNA-seq profiles and survival data for analysis. We installed a 3:2 ratio to randomly group these samples as the training cohort (n = 315) and internal validation cohort (*n* = 208). Furthermore, expression profiling and clinical information on the validation cohorts were downloaded from the ICGC and E-MTAB databases. All data were processed through R (version 4.0.3) and converted to FPKM value format. In addition, we obtained eight ccRCC datasets from the GEO database, transformed the data to the Z score, and validated the expression levels of KNCJ16. We built an outline diagram for the whole process of the study (Figure 1).

**TABLE 1:** Clinical information of 530 ccRCC patients.

Clinical parameters	Variable	Total (530)	Percentages (%)
Age	≤60	263	49.62
	>60	267	50.38
Gender	Male	344	64.91
	Female	186	35.09
AJCC stage	Stage I	265	50.00
	Stage II	57	10.75
	Stage III	123	23.21
	Stage IV	83	15.66
	Unknown	2	0.38
ISUP grade	G1	14	2.64
	G2	227	42.83
	G3	206	38.87
	G4	75	14.15
	GX	8	1.51
Survival status	Dead	173	32.64
	Alive	357	67.36

### Identification of Differentially Expressed and Prognostic Ion Channel-Related Genes

We first screened and documented ion channel-related genes (ICRGs) through an extensive literature review. The detailed list of the ICRGs is given in Supplementary Table S1. The differentially expressed genes (DEGs) between tumor and normal samples were screened using R studio’s “Limma” package. |Log2FC| > 1, *p* < 0.05, and false discovery rate (FDR) < 0.05 were set as the cutoffs for the DEGs in both TCGA and ICGC cohorts. An interaction network between the DEGs was mapped by GeneMANIA website (Warde-Farley et al., 2010). The DEGs that were significant in both cohorts were used for subsequent analysis. Univariate Cox regression was used to identify the prognostic ICRGs. Genes with a *p*-value less than .05 in Cox regression were identified as prognostic genes for further LASSO regression analysis.

### Construction and Validation of the Diagnostic Signature

Based on the previously obtained DEGs, we used both the least absolute shrinkage and selection operator (LASSO) and support vector machine recursive feature elimination (SVM-REF) to identify diagnostic models in ccRCC and normal tissues (Xia et al., 2020). The genes obtained by the two methods were taken as intersections and validated with ROC curves. The expression of intersecting differential genes in ccRCC and normal tissues was also explored in the Human Protein Atlas (HPA) database.

### Molecular Subtyping and Therapeutic Prediction

To subtype patients and thus proceed with individualized treatment, we performed molecular subtyping of patients in TCGA based on prognostic ICRGs using the ConsensusClusterPlus package (Wilkerson and Hayes 2010). The RNA matrix was calculated using the k-means algorithm on a fraction of probes (0.8) with 1,000 repeats. Meanwhile, unsupervised clustering and corresponding representative figures were generated with the ggplot2 package (Ito and Murphy 2013). Then, a single-sample Gene Set Enrichment Analysis (ssGSEA) was performed to identify immune cell infiltration between the two clusters. The potential molecular enrichment of the two clusters was annotated with the ClueGO plugin (Bindea et al., 2009). The pRRophetic algorithm was performed to calculate the half-maximal inhibitory concentration (IC50) of chemotherapeutic drugs in clusters (Geeleher et al., 2014). At the same time, we applied The Cancer Immunome Atlas (TCIA) database to observe the response to immunotherapy between the two clusters.

### Gene Pathway Enrichment Analyses

The pathway enrichment analyses were conducted using the ClusterProfiler packages to explore the potential functions, and the Gene Ontology (GO) and Kyoto Encyclopedia of Genes and Genomes (KEGG) were chosen as the background databases (Yu et al., 2012). The Gene Ontology (GO) analysis identified biological processes (BPs), molecular functions (MFs), and cell components (CCs) enriched for each set of genes.

### Construction and Validation of the Prognostic Signature

To assess the value of ICRGs as a guide to the prognosis of ccRCC, we first divided the samples in TCGA into a training and validation set on a 3:2 basis while employing the E-MTAB-1980 database as an external validation set. Furthermore, we trained the prognostic ICRGs on LASSO with maxit = 1,000 and constructed a 10-gene prognostic model. ROC curves and K–M survival plots were condemned to assess the predictive value of prognostic models using survival and time ROC packages.

### Establishment of the ICRG-Based Nomogram

To better assess clinical prognosis and provide timely intervention, we combined risk scores and clinical parameters to construct the nomogram. The “rms” package was used to establish the nomogram of multivariable models for 1-, 3-, and 5-year OS prediction. Furthermore, the calibration curve was carried out for the constructed nomogram, and the discriminative performance is quantified using the area under the curve (AUC).

### Statistical Analysis

In the results of this study, continuous variables are described by mean ± S.D, while categorical variables are characterized by frequency (n) and proportion (%). Univariate and multivariate Cox regression analyses were used to detect independent factors. The survival analysis evaluated the correlation between the characteristics and overall survival by LASSO regression models. Kaplan–Meier survival curves and time-dependent ROC curves were drawn and compared among subgroups. In addition, statistical analyses were performed using R version 4.0.3 and GraphPad 9.0. A *p*-value of less than .05 was considered statistically significant.

## Results

### Identification of Differentially Expressed Ion Channel-Related Genes

**FIGURE 2:**
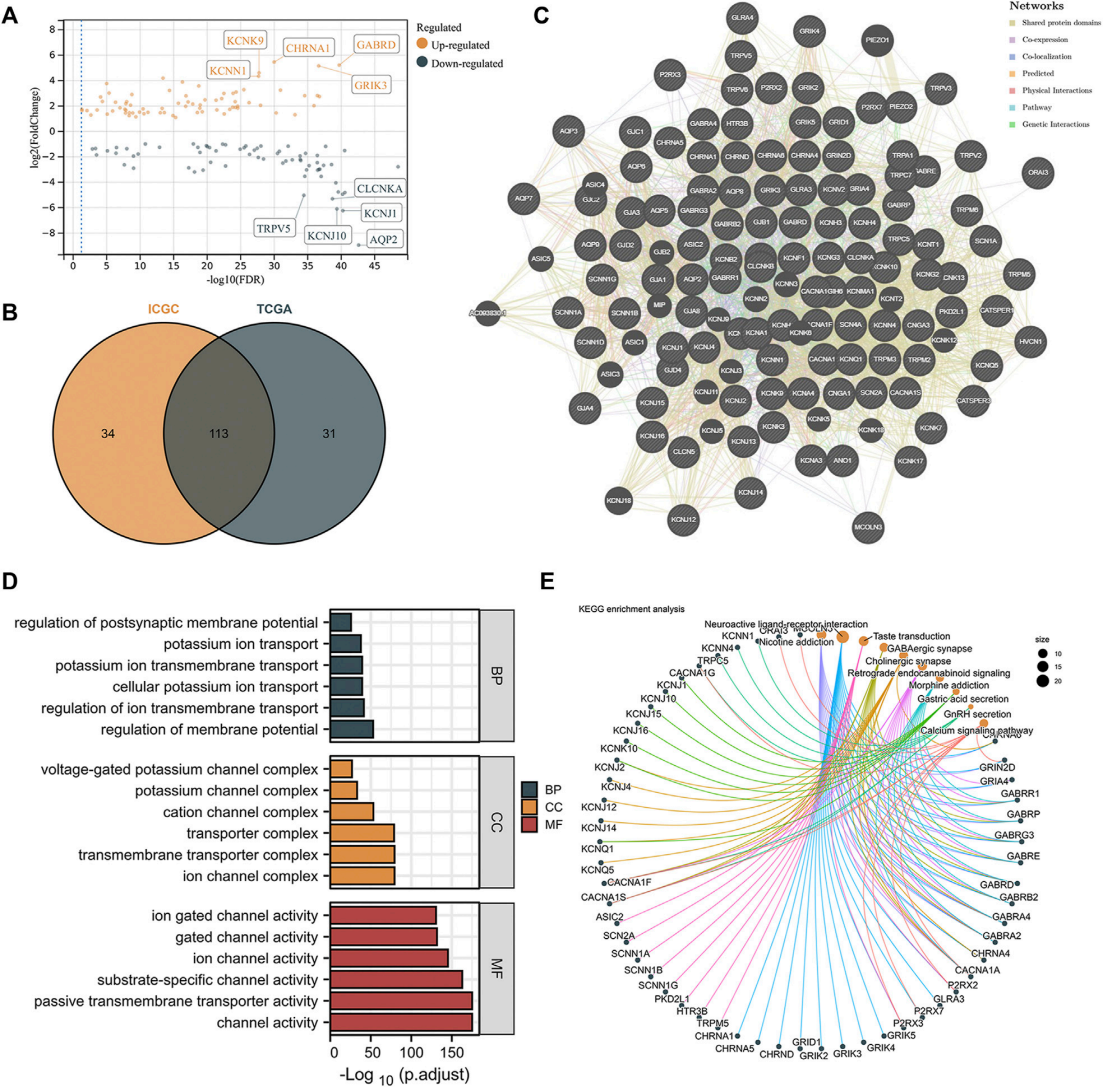
Identification and functional enrichment of differentially expressed ICRGs. **(A)** Volcano map on differentially expressed ICRGs in TCGA cohort. The five most significant up- and downregulated genes were labeled separately. **(B)** VENN plots of the same differential genes in ICGC and TCGA databases. **(C)** PPI network created by GeneMANIA showing the interactions of the ICRGs. **(D)** Bar plot for GO pathways in TCGA cohort (the longer bar means the more significantly enrichment). **(E)** Circle diagram of the KEGG pathway analysis (larger pathway point size indicates more IRCGs are included).

The expression levels of ICRGs were analyzed between ccRCC and normal samples in both TCGA and ICGC databases with a *p*-value < 0.05 and absolute log_2_FoldChange >1. The volcano plot exhibited the DEGs in TCGA and identified the five most significantly up- and downregulated genes (Figure 2A). Afterward, we considered 113 genes altered considerably in both datasets as DEGs in ccRCC (Figure 2B). Subsequently, to understand the correlation between these differential genes, we constructed a gene interaction network by GENEMANIA (Figure 2C). The interaction network demonstrated a variety of potential connections between DEGs, including potential interaction links such as co-expression and co-localization. Then, the GO enrichment analysis showed an enrichment of GO terms indicative of functional maturation, such as ion transport or ion channel composition, which is consistent with the function of these ICGRs (Figure 2D). In addition, the analysis based on KEGG pathways identified several significant enrichments, such as nicotine addiction and the calcium signaling pathway (Figure 2E).

### Construction and Validation of the Diagnostic Signature

**FIGURE 3:**
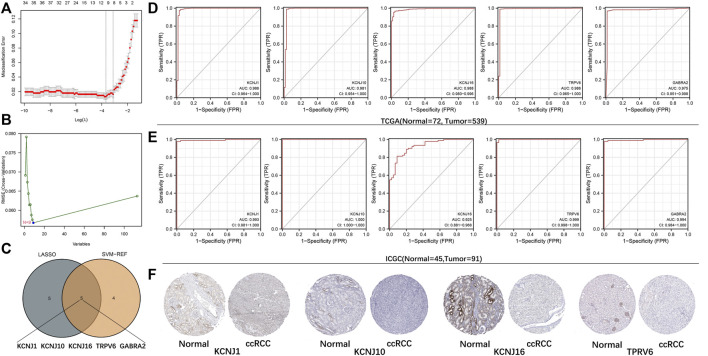
Establishment and validation of the diagnostic signature based on ICRGs. **(A)** LASSO regression to identify signature genes in ccRCC and normal samples. **(B)** SVM-REF approach to identify signature genes in ccRCC and normal samples. **(C)** Venn diagram based on the intersection of the two algorithms with five genes. **(D)** ROC curves of five signature genes for predicting the diagnostic value in TCGA cohort. **(E)** ROC curves of five signature genes for predicting the diagnostic value in the ICGC cohort. **(F)** Expression trends of four signature genes (except for GABRA2) were observed in protein expression data based on the HPA database.

The DEGs were obtained and their potential associations and enriched pathways were analyzed. Next, we explored whether ICRGs could be used as a diagnostic model to determine ccRCC. We screened 10 and nine ICRGs by LASSO regression and SVM-REF analyses, respectively (Figures 3A, B). We then selected the intersecting genes of the two methods as diagnostically relevant genes, which were KCNJ1, KCNJ10, KCNJ16, TRPV6, and GABRA2 (Figure 3C). Subsequently, we evaluated the predictive diagnostic value of these five ICRGs by AUC values in TCGA and ICGC databases, and the results showed a good predictive value in ccRCC or RCC (Figures 3D, E). Subsequently, we explored the protein expression levels of these genes in the HPA database. It could be seen that KCNJ1, KCNJ10, KCNJ16, and TPRV6 expression levels were all significantly upregulated in normal tissues compared with tumor tissues and distributed in different normal tissue structures (Figure 3F). Due to the lack of relevant IHC staining for GABRA2 in the database, the IHC image of GABRA2 has not been exhibited. Corresponding informations has been changed in the manuscript too.

### Molecular Subtyping and Therapeutic Target Screening

**FIGURE 4:**
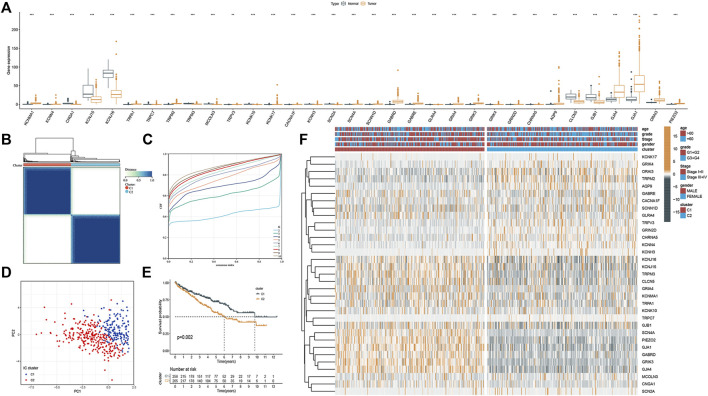
Molecular subtyping based on the ICRGs in TCGA cohort. **(A)** Box plot of the 33 DEGs between tumor and normal samples. **(B)** Total of 374 ccRCC patients were identified into two clusters according to the consensus clustering matrix (k = 2). **(C)** Relative changes in the area under the CDF curve by group number (MaxK = 10). **(D)** Principal component analysis on the two clusters based on the 33 DEGs. **(E)** Kaplan–Meier survival curves for the two clusters (*p* = 0.002). **(F)** Heatmap and the clinical parameters of the two clusters established by 33 DEGs (the more yellow the color, the higher the level of gene expression. The bluer the color, the lower the level of gene expression.).

Molecular subtyping could provide insights into tumor biology for potential therapeutic targets. So, in this section, we explored whether ICRGs could be used in molecular subtyping for ccRCC patients. First, by performing the Univariate Cox analysis, we screened out 33 ICRGs significantly associated with OS and defined them as prognostic ICGRs. The mRNA expression level of 33 prognostic ICRGs between ccRCC and normal samples was exhibited (Figure 4A). We then conducted the ConsensusClusterPlus package and identified two clusters (C1 and C2), according to the consensus distribution function plot (Figures 4B, C). Principal component analysis (PCA) showed good separation between the two classifications and the survival showed a worse prognosis for patients in C2 than in C1. We then drew a heatmap of the composite clinical information based on the clusters. The gene expression differences between the two clusters and the corresponding clinical grading and staging distribution can be apparently shown (Figure 4E).

### Phenotypic Differences Between Clusters and Potential Individualized Treatment

**FIGURE 5:**
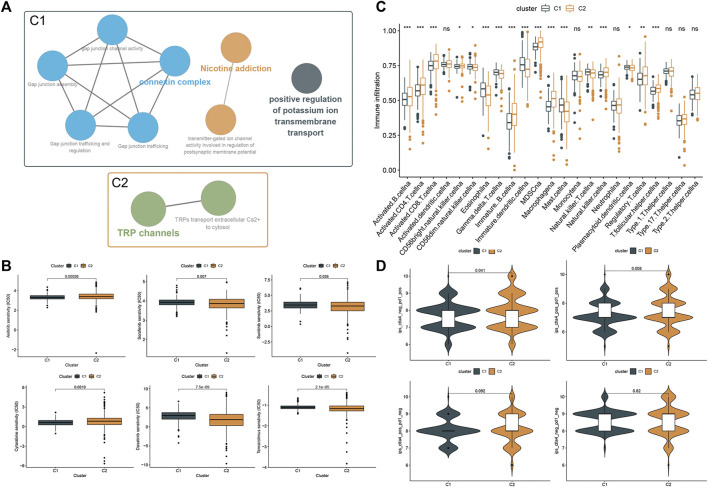
Phenotypic differences between clusters and potential individualized treatment. **(A)** Biological functional annotation of differentially expressed genes between two clusters. **(B)** Drug sensitivity of commonly clinically used drugs for advanced ccRCC between clusters. **(C)** Differential analysis of immune cell infiltration between the two clusters using the ssGSEA method. **(D)** Relationship between differences in PD-1 and CTLA-4 responsiveness between the two groups, based on the TCIA database.

To explore potential features between the two clusters, we identified gene expression differences between the clusters by using the limma package and performed functional enrichment using the ClueGO plugin. We found that patients in cluster one were significantly associated with connexin complex, nicotine addiction, and positive regulation of potassium ion transmembrane transport, while patients in cluster two were significantly correlated with TRP channels (Figure 5A). Subsequently, we explored the sensitivity of the two clusters to drugs commonly used in clinically advanced ccRCC by pRRophetic. The results showed that the patients in cluster1 were more sensitive to Axitinib and Cytarabine, while the patients in cluster 2 were more sensitive to the remaining four drugs (Figure 5B). Subsequently, differences in immune cell infiltration were explored between our two clusters. Immune cell infiltration was more abundant in cluster 2 (Figure 5C). Also, we explored the responsiveness to immunotherapy in the TCIA database. More patients in cluster two were PD-1 positive or PD-1/CTLA-4 double positive and may have benefited more from immunotherapy (Figure 5D).

### Construction and Validation of the Prognostic Signature

**FIGURE 6:**
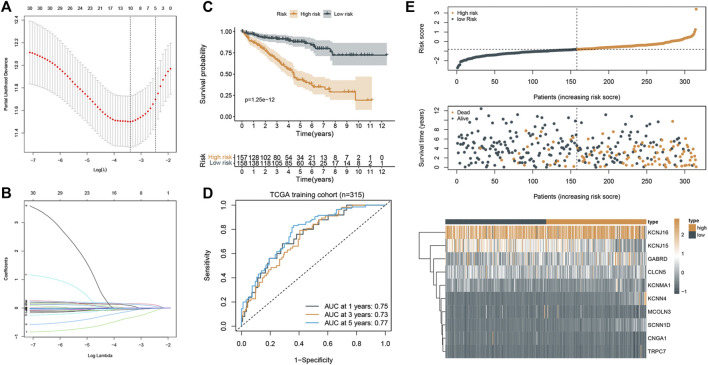
Construction and validation of the prognostic signature. **(A)** Cross-validation of the parameter selection in the LASSO regression. **(B)** LASSO regression of the 10 ICRGs related to the OS. **(C)** Kaplan–Meier survival curves between high- and low-risk groups (*p* < 0.001). **(D)** ROC curves of the prognostic signature for predicting 1-year, 3-year, and 5-year OS in TCGA cohort. **(E)** Signature gene expression patterns and the distribution of survival status and risk score in TCGA training cohort.

Based on the aforementioned prognostic ICRGs, we further evaluated the prognostic value of ICRGs in ccRCC patients. The model coefficients were solved by LASSO regression (Figure 6A, B). The detailed information on the ICRGs from the signature is shown in Table 2. The risk score is constructed according to the following formula mentioned in the Methods section. Based on the calculated median risk score cut-off, patients were divided into high- and low-risk groups. Furthermore, the Kaplan–Meier log-rank test and the time-dependent ROC curve were used to evaluate the predictive ability and accuracy of the prognostic signature. The outcome of the Kaplan–Meier log-rank test showed that the high-risk group had a significantly worse OS compared with the low-risk group in TCGA training set (Figure 6C), TCGA validation set (Supplementary Figure S1A), and E-MTAB validation set (Supplementary Figure S1C). Meanwhile, the time-dependent ROC curve proved the 1-, 3-, and 5-year predictive accuracy of the signature for OS (Figure 6D; Supplementary Figures S1B, D). In addition, the risk score distribution, survival status, and expression of ICRGs from the signatures are exhibited in TCGA training set, TCGA validation set, and E-MTAB validation set (Figure 6E; Supplementary Figures S1E, F).

**TABLE 2:** Detailed information of the ICRGs in the prognostic signature.

Gene	Ensemble ID	Description	Located	Coef
KCNMA1	ENSG00000156113	Potassium calcium-activated channel subfamily M alpha 1	10q22.3	−0.05348
KCNN4	ENSG00000104783	Potassium calcium-activated channel subfamily N member 4	19q13.31	0.148,382
CNGA1	ENSG00000198515	Cyclic nucleotide gated channel subunit alpha 1	4p12	−0.34209
KCNJ15	ENSG00000157551	Potassium inwardly rectifying channel subfamily J member 15	21q22.13	−0.02291
KCNJ16	ENSG00000153822	Potassium inwardly rectifying channel subfamily J member 16	3q13.2	−0.00794
TRPC7	ENSG00000069018	Transient receptor potential cation channel subfamily C member 7	5q31.1	0.084228
MCOLN3	ENSG00000055732	Mucolipin TRP cation channel 3	1p22.3	-0.10732
SCNN1D	ENSG00000162572	Sodium channel epithelial 1 subunit delta	1p36.33	0.135,559
GABRD	ENSG00000187730	Gamma-aminobutyric acid type A receptor subunit delta	1p36.33	−0.00837
CLCN5	ENSG00000171365	Chloride voltage-gated channel 5	Xp11.23	-0.00482

### Construction and Validation of the Nomogram

**FIGURE 7:**
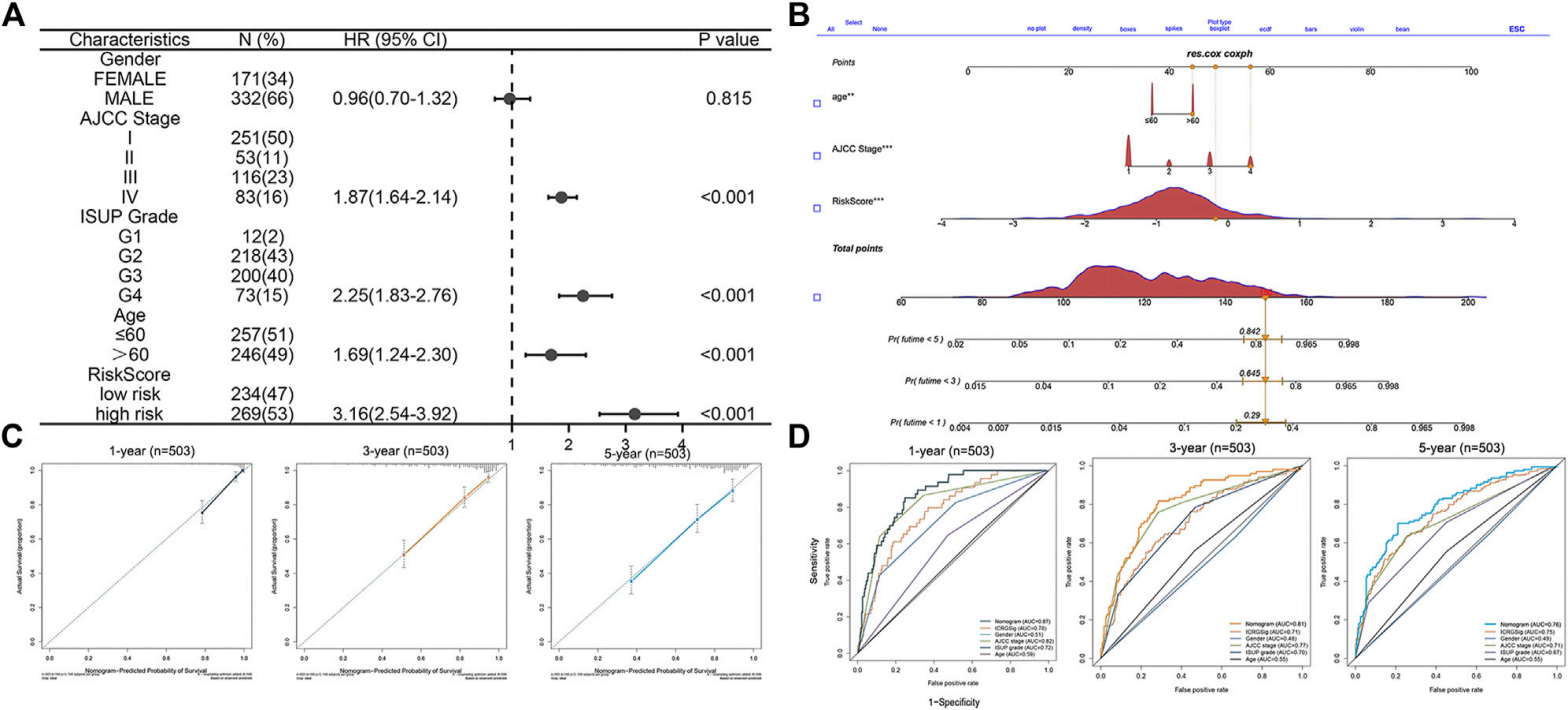
Construction and validation of the prognostic nomogram. **(A)** Forest plot of the significant clinical parameters in the univariate Cox regression. **(B)** Nomogram based on the significant clinical parameters and risk signatures. **(C)** Calibration curves of the nomogram for 1-, 3-, and 5-year survival prediction. **(D)** Predictive value of the nomogram, risk signature, and clinical parameters.

To better predict the prognosis of patients, we combined clinical factors and risk signatures to construct the nomogram. Univariate Cox regression analysis was performed to screen out the significant factors with OS (Figure 7A). Afterward, multivariate Cox regression analysis showed that the AJCC stage, age, and risk score could be the independent factors for OS of ccRCC patients (Table 3). Combining the risk score and clinical parameters, we established the nomogram with a C-index of 0.774 (Figure 7B). Then, we performed the calibration curves to verify the predictive efficacy of the nomogram for 1-, 3-, and 5-year OS (Figure 7C). We also performed ROC curves, and the AUC values indicated that the predictive efficacy of the nomogram was significantly better than that of the present AJCC stage or ISUP grade and the prognostic signature alone (Figure 7D).

**TABLE 3:** Univariate and multivariate Cox analyses of clinical parameters and risk signatures.

Parameters	Univariate analysis		Multivariate analysis	
	HR (95% CI)	*p* Value	HR (95% CI)	*p* Value
Gender	0.963 (0.703, 1.319)	0.815	1.023 (0.740, 1.326)	0.886
AJCC stage	1.870 (1.638, 2.136)	<0.001	1.529 (1.310, 1.784)	<0.001
ISUP grade	2.251 (1.835, 2.763)	<0.001	1.233 (0.971, 1.565)	0.086
Age	1.690 (1.241, 2.303)	<0.001	1.588 (1.161, 2.172)	0.003
ICRGSig	3.157 (2.563, 3.917)	<0.001	2.170 (1.694, 2.781)	<0.001

### Further Exploration of the KCNJ16 Gene

**FIGURE 8:**
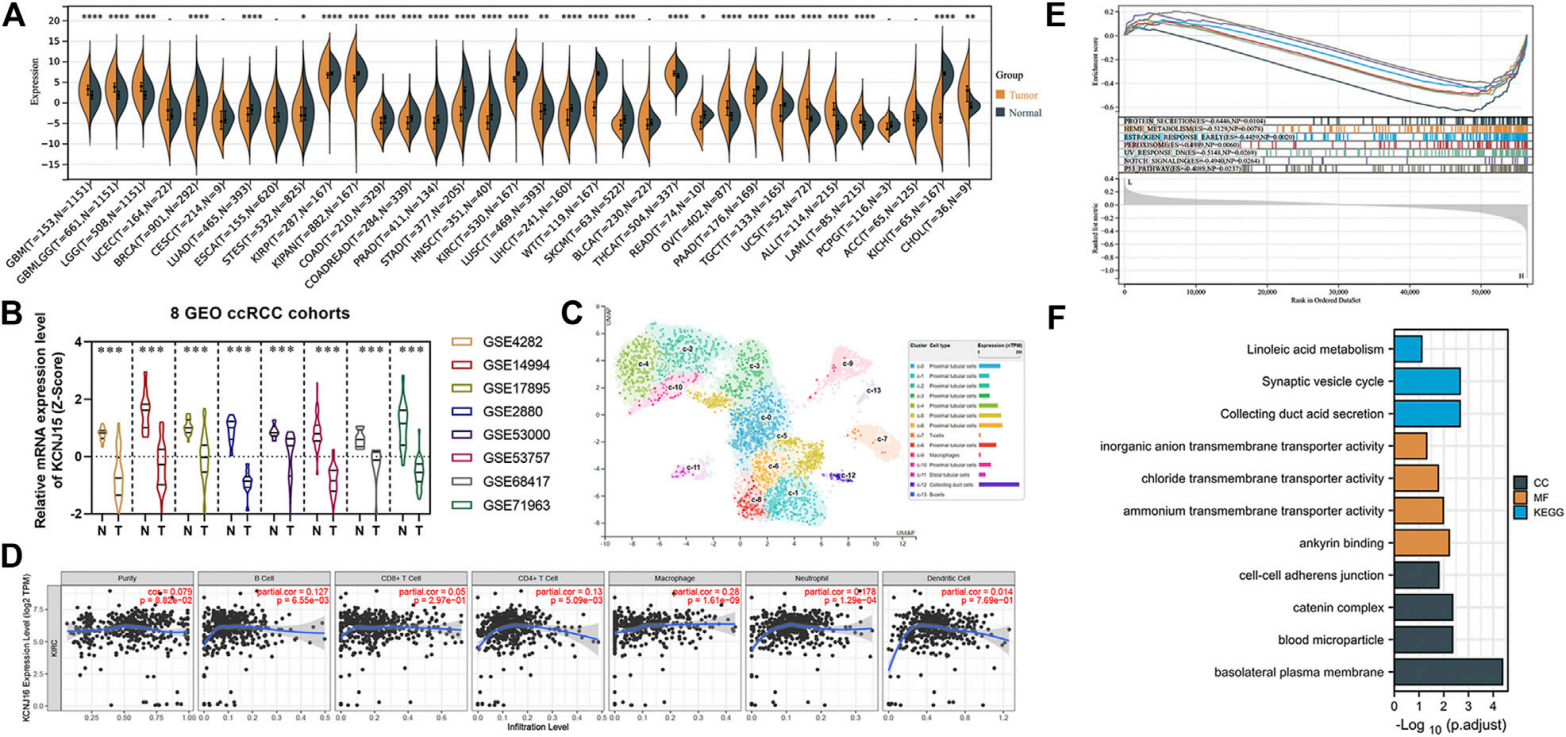
Further exploration of the KCNJ16. **(A)** Pan-cancer analysis of the KCNJ16 mRNA expression among 33 types of cancers. **(B)** Analysis of eight GEO datasets regarding KCNJ16 expression in ccRCC and normal samples. **(C)** Single-cell analysis of the KCNJ16 expression in kidney tissues. **(D)** Association of the KCNJ16 expression level with immune infiltration abundance in ccRCC was evaluated using the TIMER database. **(E)** Multiple GSEA analysis on potential biological mechanisms between low- and high-risk groups. **(F)** GO and KEGG enrichment analyses between high- and low-risk groups.

We then analyzed the KNCJ16 gene, an ICRG that we had previously derived to be significant in both diagnostic and predictive models. First, we found that the KCNJ16 expression level was significantly altered in a variety of cancers by pan-cancer analysis, using the SangerBox and was more apparent in ccRCC (Figure 8A). We then compared the expression levels of KCNJ16 in cancer and normal tissues in eight GEO databases and found that the expression levels of KNCJ16 were significantly upregulated in normal tissues (Figure 8B). Based on the aforementioned results, we wanted to clarify exactly in which structure of the kidney that the KCNJ16 expression levels are upregulated, and we explored this with single-cell data from the HPA database. We found that the KCNJ16 gene was mainly upregulated in the proximal tubular cells and collecting duct cells (Figure 8C). Meanwhile, we screened out the relationship between KNCJ16 and immune cell infiltration using the TIMER database. Consistent with the HPA database, the correlation between KCNJ16, T cells, and B cells was not significant, while mainly affecting macrophage components (Figure 8D). Furthermore, we explored the potential biological functions of KCNJ16. GSEA enrichment analyses showed that high KCNJ16 expression was significantly associated with protein secretion, p53 pathway, and so on (Figure 8E). Meanwhile, GO and KEGG enrichment analyses indicated that KCNJ16 was closely related to the transmembrane transport function and cell structure maintenance (Figure 8F).

## Discussion

Early detection and prognostic evaluation of ccRCC is crucial, however, there were certain shortcomings in either imaging or the tumor-node-metastasis (TNM) staging system at present (Jonasch et al., 2021). Finding suitable biomarkers is of utmost importance. As an important organ for nutrient absorption and substance metabolism, numerous ion channels and corresponding proteins are widely distributed in the kidney (Valinsky et al., 2018; Su et al., 2020). Meanwhile, during the development of ccRCC, the destruction of a large number of normal structures makes ion channels possible detective and prognostic markers of kidney carcinogenesis and progression. Therefore, using the ICRGs for the diagnosis and evaluation of ccRCC might be promising.

Previous reports have shown that ICRGs may play important roles in ccRCC. Some ICRGs have been reported to be aberrantly expressed in ccRCC and were closely correlated with the prognosis of ccRCC patients (Rabjerg et al., 2015; Lkhagvadorj et al., 2016; Xu et al., 2019). TRPM7 can regulate tumor growth by modulating the AKT/FOXO1 axis (Zhao et al., 2018). TRPM3, on the other hand, affects the tumor progression of ccRCC by regulating autophagy (Hall et al., 2014). Interestingly, due to the imbalance between the tumor proliferation rate and oxygen supply, there is widespread hypoxia, and consequently, lactate accumulation in solid tumors. ICRGs can modulate the tumor microenvironment through the action of ion channels, which in turn affects immune cell infiltration and immunotherapy (Gerlinger et al., 2012; Muz et al., 2015; Li et al., 2015). Hence, in this study, we systematically explored the diagnostic and prognostic value of ICRGs through multiple ccRCC databases and established the corresponding signature. Then, we evaluated immune infiltration and potential individualized therapeutic targets by establishing molecular subtyping.

We first screened five ICRGs that were closely associated with the diagnosis of ccRCC by the SVM-REF and LASSO methods. With the exception of GABRA2, the remaining four genes are thought to be markers for certain specificities in normal kidney tissues (Nijenhuis et al., 2003; Welling 2016). Consistent with our previous speculation, the massive destruction of normal tissues during the development of ccRCC leads to abnormal alterations of these marker genes. Detection of the expression of these marker genes might provide a new insight for the diagnosis of ccRCC. Furthermore, previous studies have revealed that Kir5.1 and Kir4.1 potassium channels played important roles in the immune regulation of tuberous sclerosis (Schirmer et al., 2014). In the kidney, the proteins that make up these potassium channels can be used as diagnostic markers, which may be an interesting phenomenon. Subsequently, we screened the ICRGs that were significantly associated with OS by Cox regression and identified them as prognostic ICRGs. We then divided the samples in TCGA into two clusters by consensus clustering, which showed significant differences between the two clusters. We then analyzed immune infiltration and efficacy against immune checkpoint therapy between the two clusters. Patients in C1 might be more sensitive to treatment with PD-1 and CTLA-4. Meanwhile, for the present clinical application of TKIs drugs for the treatment of advanced ccRCC, we explored the sensitivity of patients to these drugs in the two clusters through the GDSC database. We found that C1 patients were more sensitive to Axitinib and Cytarabine, while C2 patients were more sensitive to Sorafenib, Dasatinib, Temsirolimus, and Sunitinib. This difference in sensitivity to drugs can provide some reference for individualized patient treatment.

We then constructed a 10-ICRG-based signature using LASSO regression (KCNMA1, KCNN4, CNGA1, KCNJ15, KCNJ16, TRPC7, MCOLN3, SCNN1D, GABRD, and CLCN5). In addition to showing better predictive performance, most of the genes have shown a close relationship with cancer in previous studies. KCNMA1 has been used as a prognosis-related biomarker in several tumors and is strongly correlated with tumor metastasis and calcium channels (Khaitan et al., 2009; Zhang and Yan 2014; Wang et al., 2018). GABRD is widely reported to be closely associated with colon cancer (Niu et al., 2020; Wu et al., 2020). KCNJ15 has been closely related to potassium channels and identified as a molecular marker by investigators in ccRCC (Liu et al., 2019; Zhang J. et al., 2021). Furthermore, we also focused on exploring KCNJ16, which was significant in both diagnostic and prognostic signatures. Previous studies have shown that KCNJ16 is a component protein of the Kir5.1 potassium channel, which regulates pH and responds to hypoxia *in vivo*. Interestingly, KCNJ16 is highly expressed in the renal tubule and collecting duct system, and renal clear cell carcinoma occurs in these sites (Zhang Y. et al., 2021). This indicated that KCNJ16 expression played an important role in the development of ccRCC. Moreover, the TIMER database shows a high correlation between KCNJ16 and macrophage infiltration. Previous studies suggested that the activation of potassium channels could affect the immune function of macrophages via NLRP3 (Drinkall et al., 2022). In contrast, macrophages can reshape the tumor microenvironment of ccRCC and thus influence tumor immunity (Koh et al., 2021; Dong et al., 2022). There might be some important associations between KCJN16 regulation of potassium channels and macrophages affecting tumor immunity. However, its role in ccRCC remains to be explored.

There are some shortcomings in this study. First of all, our study is based on various published databases, and it was difficult to completely eliminate these effects because of batch effects and differences between the databases. Second, our sample size is still small, which inevitably constrained the prediction performance because of the limitation of sample size when performing model training and validation. Finally, some of the genes we identified need further validation *in vitro* and *in vivo*, such as KCNJ16.

## Conclusion

In conclusion, we were the first to systematically elucidate the diagnostic and prognostic value of ICRGs. Through prognostic ICRGs, molecular subtyping and drug sensitivity exploration was performed for patients in different clusters. In this study, we established useful signatures for early detection, prognosis evaluation, and individualized treatment for ccRCC.

## Data Availability

The original contributions presented in the study are included in the article/Supplementary Material; further inquiries can be directed to the corresponding author.
