# Supplementary figures and images for: The Ion Channel-Related Gene Signatures Correlated With Diagnosis, Prognosis, and Individualized Treatment in Patients With Clear Cell Renal Cell Carcinoma
Source: Front Pharmacol. 2022 Jun 1;13:889142. doi: 10.3389/fphar.2022.889142 (PMC9198310; doi:10.3389/fphar.2022.889142)

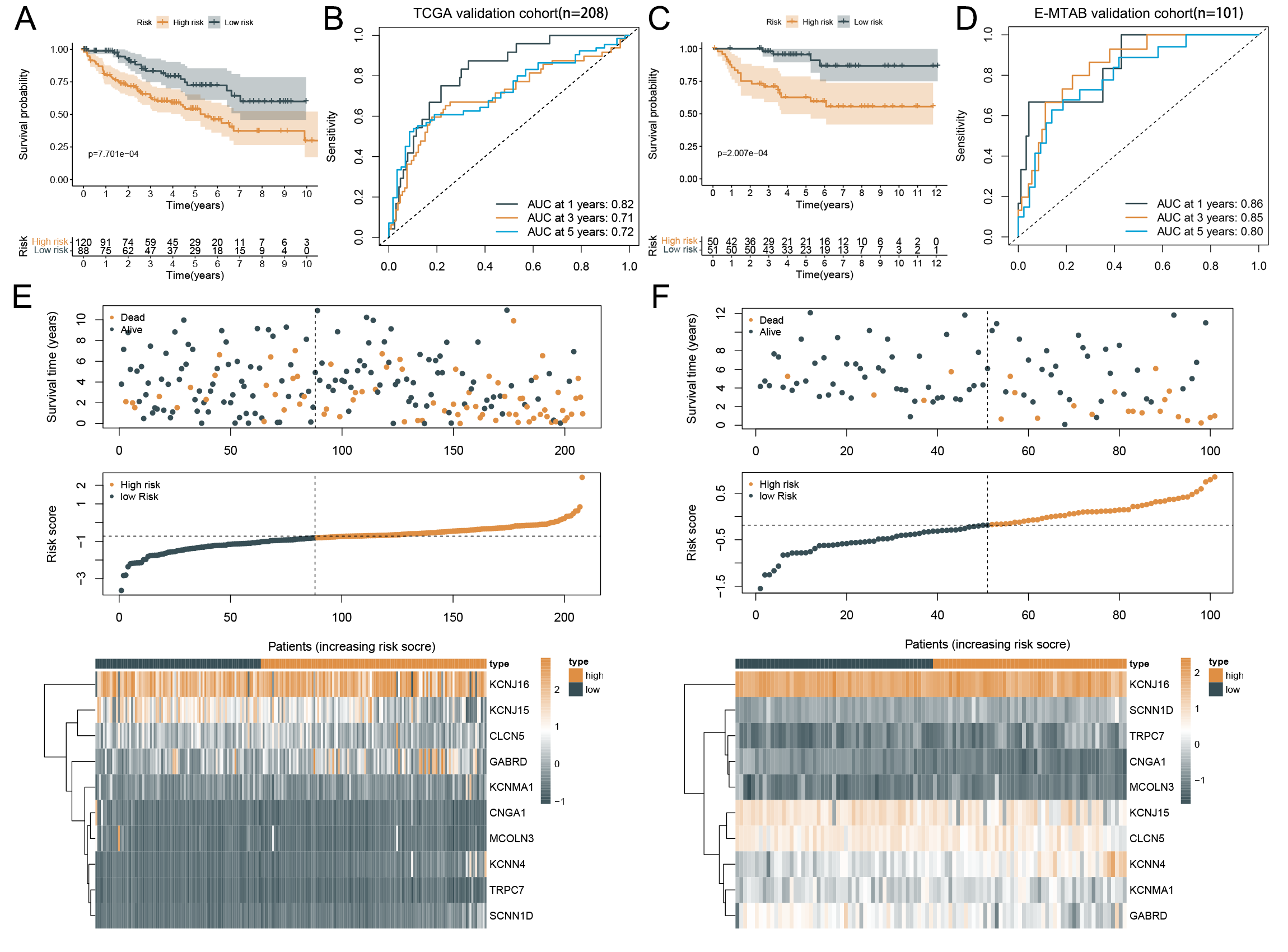

Supplement: Supplementary file 1 [file Image1.TIF]
